# From Acellular Matrices to Smart Polymers: Degradable Scaffolds that are Transforming the Shape of Urethral Tissue Engineering

**DOI:** 10.3390/ijms20071763

**Published:** 2019-04-10

**Authors:** Tariq O. Abbas, Huseyin C. Yalcin, Cristian P. Pennisi

**Affiliations:** 1 Laboratory for Stem Cell Research, Department of Health Science and Technology, Aalborg University, 9220 Aalborg, Denmark; cpennisi@hst.aau.dk; 2 Pediatric Surgery Department, Hamad General Hospital, 3050 Doha, Qatar; 3 College of Medicine, Qatar University, 2713 Doha, Qatar; 4 Surgery Department, Weill Cornell Medicine–Qatar, 24144 Doha, Qatar; 5 Biomedical Research Center, Qatar University, 2713 Doha, Qatar; hyalcin@qu.edu.qa

**Keywords:** urethral strictures, urethral tissue engineering, biodegradable polymers, acellular matrix, smart polymers

## Abstract

Several congenital and acquired conditions may result in severe narrowing of the urethra in men, which represent an ongoing surgical challenge and a significant burden on both health and quality of life. In the field of urethral reconstruction, tissue engineering has emerged as a promising alternative to overcome some of the limitations associated with autologous tissue grafts. In this direction, preclinical as well as clinical studies, have shown that degradable scaffolds are able to restore the normal urethral architecture, supporting neo-vascularization and stratification of the tissue. While a wide variety of degradable biomaterials are under scrutiny, such as decellularized matrices, natural, and synthetic polymers, the search for scaffold materials that could fulfill the clinical performance requirements continues. In this article, we discuss the design requirements of the scaffold that appear to be crucial to better resemble the structural, physical, and biological properties of the native urethra and are expected to support an adequate recovery of the urethral function. In this context, we review the biological performance of the degradable polymers currently applied for urethral reconstruction and outline the perspectives on novel functional polymers, which could find application in the design of customized urethral constructs.

## 1. Introduction

The male urethra can be affected by several primary, as well as secondary conditions, including hypospadias, fistulas, trauma, and malignancy [1]. In children, hypospadias is a common congenital malformation of the external genitalia, where shortage of the urethra is a major concern [2]. This disorder occurs in about 1 out of every 200 live male births and its surgical management remains challenging, especially in the severe forms where the patients suffer from post-operative complications [3,4]. In adult males, urethral injuries can take place at any part of the urethra distal to the bladder neck, as the result of different pathologies, including inflammatory, traumatic, or iatrogenic, with an incidence of 5000 new cases annually in the USA alone [5,6]. While male urethral conditions represent a significant medical and surgical burden, female urethral disorders are less demanding due to different etiology and anatomical constraints. Thus, management of female urethral disorders will remain outside the scope of the current review.

Overall, disorders of the male urethra may lead to a breach of the continuity of the urethral mucosa, which results in extravasation of urine, with the possibility of subsequent inflammation and, ultimately, stricture formation. The surgical management of urethral strictures differs according to the severity and length of the affected segment. While the basic approach for short segmental defects comprises resection and end-to-end anastomosis, long defects require open urethroplasty using autologous tissue grafts. Buccal mucosa is currently the most widely used graft material for urethroplasty [7,8,9]. However, limited amount of autologous tissue and donor site morbidity remain as clinical obstacles. Patients undergoing buccal tissue harvest are at risk of suffering scarring and contracture at the donor site, persistent pain, numbness, or parotid duct injury. The overall rate of oral complications has been shown to be between 3% to 4% [10]. Furthermore, buccal grafts are associated with higher costs of urethroplasty and a longer hospital stay [11].

Urethral tissue engineering has recently emerged as a viable alternative to overcome the issues associated with autologous grafts [12]. In addition to eliminating the need of harvesting autologous tissue, tissue engineered solutions are expected to become “off-the-shelf” products in the future [13]. Tissue engineering may involve scaffolds alone, which exploit the body’s natural regeneration ability, or cell-seeded scaffolds, which are used as a graft substitute. In any case, urethral tissue engineering approaches rely on biodegradable scaffold materials, which aim to recapitulate the structural, mechanical, and biological properties of the native urethra, to ultimately restore normal urethral functionality. For instance, it is desirable to provide proper mechanical compliance, to allow elastic deformation during erection of the penis, and barrier function to avoid urine leakage to the underlying tissues [14,15]. 

The aim of this article is to present the rationale for different designs for urethral replacement, as well as the limitations of each design choice. Given the inability of grafts and non-degradable polymers to meet the clinical performance requirements, this review will focus on the ability of degradable polymeric scaffolds to mimic the native urethral structure, in an effort to restore complete urethral function. Toward this purpose, we initially describe the properties of the male urethra that dictate design requirements for a completely regenerated structure. Then, we review the preclinical and clinical results obtained with current biodegradable biomaterials, discussing their advantages and limitations. Finally, we discuss the perspectives on novel functional polymers, which may find application in the field of urethral tissue engineering.

## 2. Structural and Functional Properties of the Male Urethra

**Figure 1:**
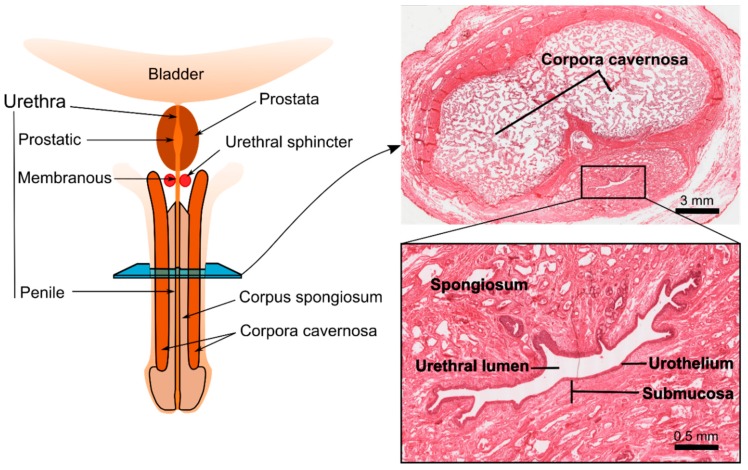
Diagram showing the general anatomical features of the human penis with a cross section at the level of the penile urethra showing the different anatomical structures. The histological examination at the level of the cross section shows the two corpora cavernosa and the urethra with its three main layers: epithelia, submucosa and spongiosum (histological images reprinted with permission from Ref. [25]). © 2019 Regents of the University of Michigan.

The male urethra is a distensible tubular structure connecting the urinary bladder to the meatus, which mainly serves as a conduit to eliminate the urine. As shown in the diagram of Figure 1, the male urethra can be divided into three segments. The most proximal segment, the prostatic urethra, passes through the prostate and constitutes the widest portion. The middle segment, the membranous urethra, is the shortest and is surrounded by the external urethral sphincter. When the sphincter is closed, the intraluminal pressure on the membranous urethra can reach up to 120 cm H_2_O. The most distal and longest portion is the penile urethra, which is surrounded by the corpus spongiosum, a richly vascularized erectile tissue that provides support to the urethra. Histologically, the urethra is composed from three different layers: an inner mucosal epithelial lining, a submucosa comprising of fibroblasts, and a muscular layer (Figure 1). The urothelial lining of the urethra appears to be essentially different from that of the bladder [16]. This can be observed by change of the epithelium from transitional (in the bladder), to pseudostratified (in the penile urethra), to stratified squamous at interface with the skin. The muscular layer is mainly composed of smooth muscle cells. The striated component extends from the base of the bladder to the membranous urethra, where it forms the external urethral sphincter for active continence. Each cell layer contains a complex extracellular matrix (ECM) structure, mainly composed of collagen I and III, glycosaminoglycans (GAGs), and elastin [17,18]. Mechanically, while collagen gives strength and structural support, elastin provides reversible extensibility [19]. While the detailed histological composition of the urethra has been thoroughly described in female animals, which are relevant models for continence research the histology of the male urethra, which can potentially be translated into urethral tissue engineering studies, has not been sufficiently explored yet [20,21,22,23,24]. 

Apart from its signaling role, to maintain the cellular homeostasis, the ECM plays an essential role in defining the mechanical compliance of the urethra, which should be able to dilate its lumen during micturition and stretch considerably during erections [26,27]. During urination, the urinary bladder’s pressure increases up to 50–60 cm H_2_O and expels the urine with a flow rate about 20–30 mL/s. One of the major points not considered frequently in producing urethral constructs is the maintenance of enough compliance of the urethra during micturition, in order to decrease patient discomfort and protect the upper urinary tracts. Different studies investigated different variables affecting urethral compliance in a clinical background [28,29,30]. Normal urethral compliance is dependent on the optimal passive viscoelastic properties of the urethral wall in order to keep the urination process under low pressure so as not to harm the urinary bladder, kidneys, and cause infections. Different therapeutic and surgical conditions of the urethra can affect these parameters and this was objectively demonstrated through in vivo experiments using different animal models [31]. We hypothetically assume that the same effects can occur when implanting tissue-engineered urethral constructs, and can be significantly ameliorated with appropriate degradability of the scaffold and subsequent healthy tissue regeneration. It is worth noting that differences in stiffness/compliance between the implanted segment (and subsequent regrown engineered neourethra and adjacent native urethra) determines the range of mechanical mismatch that still would not meet the clinical performance requirements. This reflects the inability of the compensatory capacity of the normal urethra to the noncompliant/stiff new urethral segment. This can be elicited from the clinical/functional perspective, as it is known that even a very short fibrotic segment can have as significant clinical burden as long segments. This is clear from the pathological aspects of posterior urethral valves in male children, which are the most common reason of renal transplantation before puberty. Similar outcomes are commonly observed with urethral strictures in adults, in which very short strictures can affect urinary function and represent a risk for renal complications [32].

Urethral biomechanical parameters (tension–strain relation) can explain the relation between the structure and function of the urethra. Studies have shown that the urethra possesses a nonlinear cross-sectional area pressure, meaning that the tissue is fairly deformable at low pressure (facilitating voiding), but less deformable at higher stress levels (protective against over distention) [33]. It has also been shown that abnormal urethral narrowing or obstruction, as well as post-operative complications and recurrence of pathology, can be reliably screened by uroflowmeter measurements [33,34]. Uroflowmetry is considered a sensitive noninvasive functional modality for the assessment of the lower urinary tract dynamics and could be used for better assessment of the functional outcomes. However, this structure–function relationship has not been sufficiently investigated in previous preclinical urethral tissue engineering experiments.

Adding to the complexity of the picture, in hypospadias and urethral surgery, additional biomechanical and clinical problems may arise [34,35,36]. Another aspect which is not frequently taken into account, particularly in pediatric patients, is that the urethra doubles its length during puberty. This should be a main consideration during scaffold synthesis in order to have resulting healthy tissue that have this extent of growth [37]. Urethral graft contracture often occurs following prostatectomy, hypospadias repair, cystoscopy, and urethral cauterization. Several tissue engineering approaches were examined to manage these strictures that included using tissue-engineered buccal mucosa [38]. Another approach has been exploiting the anti-inflammatory properties of stem cells, such as human bone marrow-derived mesenchymal stem cells (MSC) combined with CD34+ hematopoietic stem/progenitor cells [39].

Another important point that has not received too much attention in the design of scaffolds for urological tissue engineering is the avoidance of urine leak, even when the urine is known to be highly cytotoxic. In the normal urethra, urothelium barrier function is maintained by three structures: uroplakin proteins in the apical cell membrane, tight junctions situated between the superficial umbrella cells, and urothelial GAGs and proteoglycans, covering the umbrella cells [40]. Formation and regeneration of the urothelium are critical to prevent urethral stricture formation. In addition, an intact urothelium prevents bladder detrusor muscle over-activity [41], inflammation, and fibrosis. When the protective uroplakin barrier is dysfunctional, as it is during the early stages of urothelium regeneration, cytotoxic agents bind spontaneously to the anionic milieu of inner layers of the bladder mucosa. Thus, deep penetrating urine is believed to exert a significant impact on the viability of cells within the scaffolds [42]. Since urine has a harmful effect on the cellular components of a tissue engineered urethra, it is important that the scaffold has adequate impermeability since it might partially act as an isolation barrier. It is worth noting that leak here is not meant for fluid but for the cytotoxic components [43]. In urethroplasty, the avoidance of urine leak is supported by catheterization of the patient within the first week after graft implantation, until healthy regenerated urothelial tissue is able to provide the adequate barrier function. 

## 3. Polymeric Biomaterials in Urethral Tissue Engineering

**Figure 2:**
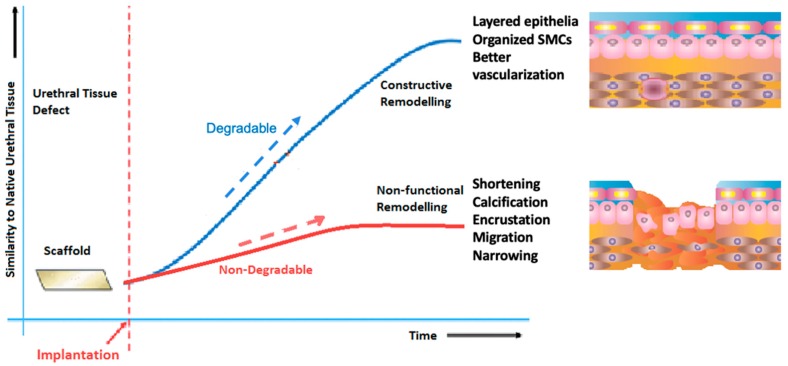
Schematic representation of the advantages of scaffold degradability with regards to restoration of “healthier” tissue engineered urethral replacement. Non-degradable biocompatible scaffolds have lead to several undesirable outcomes like shortenig of the implant, calcification, and narrowing of the urethra, collectively kown as “non-functional remodeling”. On the other hand, resulting regrown tissues following implantation of optimized degradable constructs has lead to “constructive remodeling” with the resulting tissues closer to the native normal urethra.

Initially, non-degradable materials were assessed for urinary tract reconstruction, but they resulted into several complications such as calcification, fistulae [44,45], chronic hematuria, stone formation, and development of significant contractures up to 50% of their initial lengths [46]. While non-degradable urinary scaffolds have resulted in failure of the growth of seeded cells, the addition of collagen has resulted in significant cell growth and differentiation [47]. On the other hand, degradable biopolymers are intended to permit tissue ingrowth, while the scaffold is resorbed over time, in a controlled manner (Figure 2). Physiological metabolic processes typically remove the degradation byproducts [48]. As a result, it is important to investigate the biodegradation characteristics of the scaffolds in order to achieve a successful tissue engineered replacement [49]. The incorporation of cells within the scaffold appears to improve the biocompatibility, as the cells contribute to the remodeling process through synthesis of ECM components, which are essential for the long-term survival of the implanted construct. 

The appropriate synchronization between the biodegradation of the scaffold and cellular component growth is crucial for the success of a tissue-engineered implant. The scaffold should tolerate the mechanical forces sustained during handling, suturing, and normal patient activities [50]. Biodegradable scaffolds in general have a variable biodegradation between days to several months. The timeline to allow proper function and regeneration is one of the most important variables in the design of degradable tissue engineered constructs. This actually has been explained recently in a vascular tissue engineering model of constructs, where the in vivo degradation process goes through consecutive controlled phases [51]. There is an initial “stenting phase” for merely the transfer of intraluminal fluids with minimum acute recoil, sufficient radial strength, and conformability. This is to be followed by a “restoration stage” as a result of the return of in-scaffold contractility in response to physiological (or pharmacological) stimuli, until lastly resorption occurs. Because the mechanical properties of absorbable scaffolds are designed to change over time, there are distinctive concerns for bench testing, including pretest acquisition, handling before and through testing, and time-dependent evaluations. As mentioned above, the first phase of the absorbable urethral scaffolds is to maintain lumen patency. Therefore, the essential attributes are growth capability, recoil, radial strength, and fatigue behavior. Throughout the restoration phase, the scaffold transforms from an active support of the urethra to a passive implant with a discontinuous structure. This transition is monitored by evaluating the modification in radial strength over time furthermore due to the fatigue behavior.

Degradable polymers for urethral tissue engineering have been typically obtained from natural sources or by synthesis. Natural biomaterials include acellular matrices obtained from cadaveric or animal organs via enzymatic, physical, or chemical decellularization methods, or natural polymers, such as silk fibroin (SF). In general, natural biomaterials possess integrin-binding peptide sequences and surface topography that can facilitate cellular growth and differentiation, and accelerate the process of angiogenesis [52]. Synthetic polymers, on the other hand, constitute the biggest subset biodegradable materials in use today. The most commonly used polymers include linear polyesters, poly-lactic acid (PLA), poly-glycolic acid (PGA), poly lactic-co-glycolide (PLGA), copolymers, and polycaprolactone (PCL) [53]. Some of the advantages of synthetic polymers are that they display reproducible mechanical characteristics, degrade fast, and are frequently less expensive than biologic scaffolds. However, these synthetic polymers have limited biocompatibility because they lack specific molecular elements for interaction with cells and proteins, usually requiring surface treatments to promote cell attachment [54]. Comprehensive reviews of the different biomaterials used in urethral tissue engineering are available elsewhere [12,13,55,56]. While these approaches, in general, have a goal to mimic the properties of the urethra to recover complete function, they have not been able to achieve this goal. It is, however, worth noting that it might be possible to restore the functionality of the urethra to an acceptable level without necessarily duplicating its native structure. In the following section we will focus on the preclinical results obtained using biodegradable scaffolds obtained from natural sources.

## 4. In Vivo Performance of Biodegradable Scaffolds for Urethral Reconstruction 

**Figure 3:**
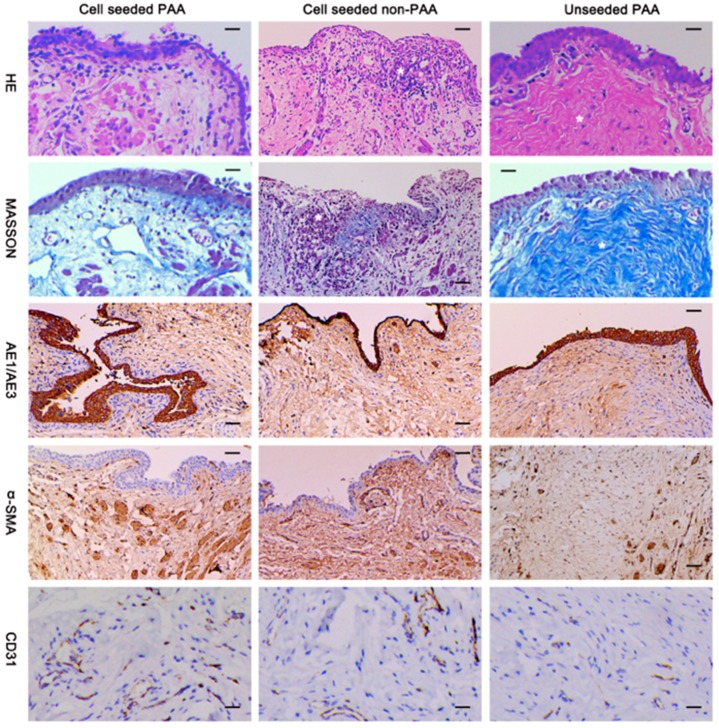
Histological and immunohistochemical assessment of urethral reconstruction in animals using cell-loaded, decellularized SIS scaffolds (Cell seeded PAA) showing regrowth of layers of transitional epithelium, more smooth muscle, and vessels in contrast to the cell loaded but non-decellularized (Cell seeded non-PAA) and non-loaded, decellularized scaffolds (Unseeded PAA). Scale bar = 20 μm. (reproduced with permission from [68]). © 2016 Zhang et al.

Acellular matrices, such as small intestinal submucosa (SIS) and bladder acellular matrix (BAM), are by far the most frequent scaffold type assessed in vivo for urethral tissue repair [57]. SIS is obtained from porcine small intestines, where the mucosal, serosal, and muscular layers are mechanically removed from the inner and outer surfaces of the intestinal wall to leave a 0.1 mm collagen-rich membrane, mostly formed of the submucosa [58,59]. In vivo animal experiments revealed that, when used for urethral replacement, SIS permitted rapid cellular growth and significant angiogenesis, often comparable to skin and mucosal grafts [60,61,62,63]. Moreover, SIS biodegrades totally in about four to eight weeks, and its degradation products are eliminated in the urine [64]. SIS has an extra benefit over other biomaterials regarding its high tendency to stretch under force and low tendency to break [65]. SIS showed favorable results when used for corporal body grafting to correct severe grades of chordee [66,67]. It was also been shown that seeding SIS with appropriate cells brings a better outcome in a rabbit model of urethroplasty (Figure 3) [68]. It is believed that extended periods of urinary diversion is important when using SIS for urethral replacement to lower the risk of urine extravasation and reduce early irritation [69]. Another option is to line it with another material having higher hydrophobicity in a multilayer manner in order to lower urine leakage. There are a number of natural material systems that have come close to restoring structure to near the native tissue. These systems, however, have still not met all the needed design constraints. The next section will describe some new materials that will help to better restore the original structure and function. Despite their evident advantages, SIS had less favorable results when used in clinical experiments, with the infection being the biggest limitation [70,71]. Furthermore, the regenerative potential of SIS depends on the age of the donor and the area of the intestine from which the SIS matrices are derived. These limitations have prevented SIS from being considered as an ideal scaffold to aid in effective urethral tissue engineering [72].

**Figure 4:**
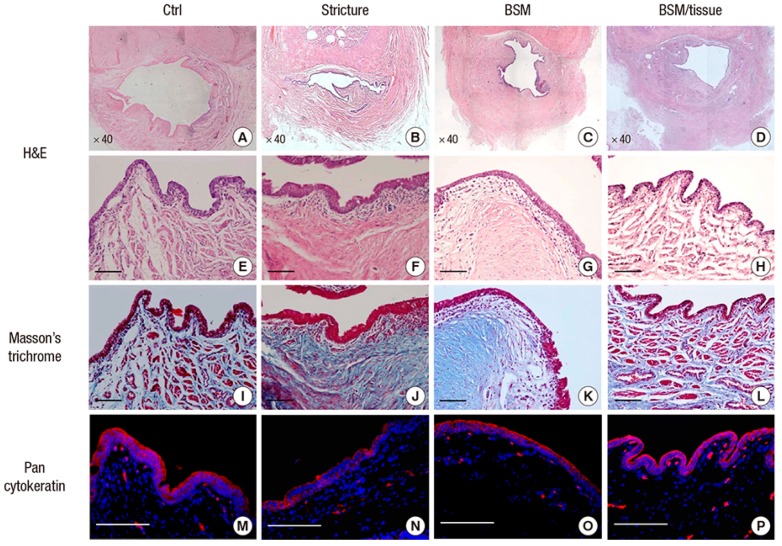
Histological and immunohistochemical analysis of urethra specimens in three groups: stricture model (one month following excision of a longitudinal strip of the urethra), ventral onlay urethroplasty repair using an acellular Bladder Submucosa scaffold (BSM), and repair using both BSM and autologous urethral tissue. It was shown that using an acellular BSM scaffold combined with an autologous urethral tissue graft resulted in a complete epithelialization, compact muscular layers, and progressive infiltration by vessels in the regenerated urethra. In contrast, the BSM grafts alone revealed keratinized epithelium, abundant collagenous fibrous connective tissue, and were devoid of bundles of circular smooth muscle. Low (**A–D**) and high-magnification (**E**–**H**) H&E images, Masson’s Trichrome images (**I**–**L**), and Pan-cytokeratin immunofluorescent images (**M**–**P**) for each of the three groups and the control (reproduced with permission from [73]).© 2015 The Korean Academy of Medical Sciences

BAM is another example of a decellularized matrix, which has shown to successfully support regeneration of the urethra in vivo [44,73,74,75,76]. Using BAM for urethroplasty in a rabbit model, implantation of cell-loaded scaffolds demonstrated a normal urethral architecture by four weeks and the neourethra could be hardly differentiated from native urethra both grossly and histologically after six months [77]. Chun et al. also showed in his experiments that ventral onlay urethroplasty using an acellular bladder submucosa matrix (BSM) scaffold combined with an autologous urethral tissue graft represents a viable option for urethral replacement (Figure 4) [73]. However, residual immunogenic components have been detected within BAM despite the stringent decellularization process [78], and further improvement in processing may be necessary for BAM to achieve successful tissue engineering. Furthermore, decellularized matrices present other limitations such as batch-to-batch variability, and may cause inflammatory reactions due to residual DNA, RNA, and xeno-antigens, which could lead to graft rejection and functional failure. 

**Figure 5:**
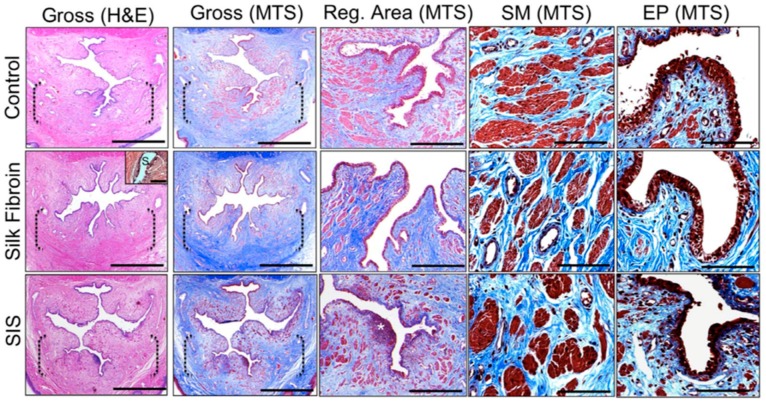
Histological analysis of urethral tissue regrowth 3 months following implantation of silk fibroin based scaffold (first and second columns). Scale bars = 3 mm. Inset: residual silk fibroin matrix fragment (S). Scale bars = 100 mm. Dashed brackets represent areas of scaffold implantation or control urethrotomy (third column). Global tissue regeneration bracketed in second column. Scale bars = 600 mm. (*) = aggregate of mononuclear cells indicative of chronic inflammation (fourth and fifth columns). Magnified de novo smooth muscle (SM) and epithelial (EP) tissue formation displayed in third column. (H&E) hematoxylin and eosin, (MTS) Masson trichrome stain. Scale bars = 200 mm. It shows that both silk fibroin and SIS scaffolds promoted similar extents of smooth muscle and epithelial tissue regeneration throughout the original defect sites with prominent contractile protein. (reproduced with permission from [86]). © 2014 Chung et al.

SF is a natural polymer derived from *Bombyx mori* cocoons that holds promise for urethral tissue engineering applications [79,80]. Traditionally, SF has been utilized as sutures because of their excellent tensile and elasticity features, in contrast to several natural and synthetic biomaterials [81,82]. SF is enzymatically degradable polymers and the degradation by-products are peptides and amino acids [83]. In comparison to SIS, SF has been shown to have less immunogenic and inflammatory responses suggesting higher levels of biocompatibility in contrast to conventional urologic biomaterials [84,85]. It was also shown that bi-layer SF scaffold for onlay urethroplasty in a rabbit model is capable of promoting similar degrees of tissue regeneration as compared to traditional SIS matrices, but with reduced immunogenicity (Figure 5) [86]. Xie et al. showed that cell-seeded SF grafts used for urethroplasty were functioning with no stricture all through the study duration (six months) [79]. 

## 5. Future Directions: Hybrid and Smart Polymers

As it was previously shown, non-degradable materials did not meet the clinical performance requirements. Despite the moderate success of degradable scaffolds in preclinical studies, the transition from bench-to-bedside remains a challenge. There are, however, some novel approaches and materials being investigated that might be able to meet the clinical performance requirements, alone or in combination with some of the other systems presented here. Natural and synthetic materials can be combined to produce hybrid biomaterials with desired properties for tissue engineering applications. Such properties include mechanical strength, porosity and cell affinity to attract cells, biocompatibility, and biodegradability for enabling replacement of ECM produced by resident cells. Current efforts in biodegradable polymer synthesis are centered on determination of appropriate biomaterials and synthesizing these materials with tailored properties for specific applications. 

Another important advancement within the present decade is the emergence of a fourth generation of “smart” biomimetic materials. These materials respond reversibly to temperature, ionic strength, pH, or light [87,88]. The responses of these polymers may include gelation, reversible adsorption on a surface, collapse of a hydrogel, and alteration between hydrophilic and hydrophobic states. Moreover, these materials can be loaded with signaling molecules like ECM components and growth factors. The delivery could be triggered using external stimuli (e.g. pH, temperature, and light) or could be done simultaneously as a result of programmable biodegradation of scaffolds [89]. Smart biomaterials have been investigated in drug delivery and medical devices application and more recently in tissue engineering applications [90]. In a broader aspect, smart biomaterials can be designed by incorporating peptide and/or protein into the polymer network, enabling the creation of a 3D scaffold for tissue engineering application [91].

Thermo-responsive polymers are the largest class of smart polymers. These are characterized with a reversibly alterable phase (or volume) transition that occurs in response to a change in temperature. The solubility of thermo-responsive polymers in aqueous solutions depend on the temperature. Above lower critical solution temperature (LCST) polymer chains are precipitated making it hydrophobic, and below LCST, polymer chains are completely hydrated, making it hydrophilic [92]. The most investigated temperature-responsive polymer with LCST in water is the poly(N-isopropylacrylamide) (PNIPAM). The LCST of PNIPAM was found to be around 32 °C, close to the human body temperature, making the polymer particularly valuable for biological applications [93]. Therefore, by changing the temperature of PNIPAM solution in water, its solubility can be changed from hydrophobic to hydrophilic and vice versa. These characteristics found application in cell sheet engineering for which cell attachment and detachment to or from material surfaces were needed. By avoiding injurious enzymes for cell detachment, cell sheets with ECM proteins could be produced [92,94]. This method might be particularly useful for producing a three-layered urethra composed of different cell monolayers (i.e. epithelial, fibroblast, and smooth muscle) [95]. In this direction, it has been shown that viable urothelial cell sheets could be advantageously obtained by using a temperature-responsive cell culture method [95].

Shape-memory polymers (SMPs) are another class of smart biomaterials. SMPs have been experimented in various vascular and bone tissue engineering/regenerative medicine studies [96,97,98]. For that reason, it has been relevant to explore the concept in response to urinary stimuli. These polymers exist in an original macroscopic shape, and temporarily stay in another shape until going back to their original shape upon exposure to a stimulus, usually heat [99]. For urethral applications, their capacity to stretch during erection and recoil during detumescence should be explored as well. Smart acellular collagen–heparin scaffolds with growth factors (GFs) were shown to support bladder tissue regeneration in a large animal model of diseased bladder [100]. Given the layered nature of urethral tissue and the need to have different cell types in a peculiar spatial distribution close to the native urethral tissue, stress-induced rolling membrane (SIRM) was shown to have the ability to potentially be able to be studied for this purpose [101].

Another subset of smart polymers that has shown promise for engineering electrically active tissues is the group of electroconductive polymers. Electroactive polyurethane polymers have shown great potential in bladder tissue engineering applications, when the aim is to regenerate muscular components and innervation [102,103,104]. Electroconductive polymers may be instrumental when applying electrical or magnetic stimulation strategies for advanced maturation of the urethral tissue engineered constructs [105]. Another potential method to enhance host–scaffold interaction is the creation of smart scaffolds, which can controllably release trophic factors to optimize host reactions toward the biomaterial [103]. 

Although several experiments have succeeded in regenerating the different urethral tissue layers (i.e., epithelial and muscular layers) [13,55,106] not much attention has been paid to the surrounding corpus spongiosum, which has been shown to be important for successful urethral surgical repair [107,108,109]. Therefore, layered scaffolds can potentially have the ability to regenerate different host tissues that resemble the normal histological architecture of the missing tissues. The incorporation of a smooth muscle cell layer has been suggested to be necessary to obtain optimal cellular grafting results [72].

**Figure 6:**
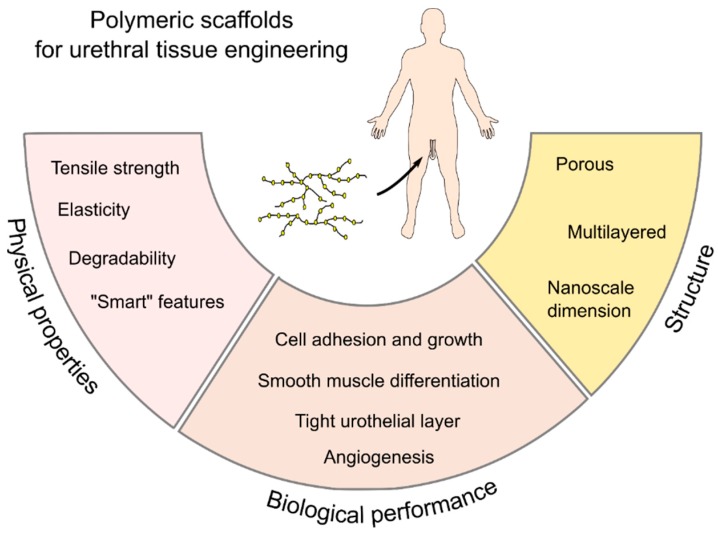
Desirable properties of polymeric scaffolds for urethral tissue engineering. These design constraints appear to be fundamental to better resemble the physical, biological, and structural properties of the native urethra and are expected to support an adequate recovery of the urethral function.

In the future, advances in understanding the physical and chemical factors responsible for urothelial and smooth muscle cell proliferation, migration, differentiation, and function may be exploited in the development of smart biomaterials, in which biologically active agents are incorporated into acellular natural or synthetic matrices. Not only interactions between urothelium and stroma, but also bi-directional interactions between tissue-engineered grafts and the host tissue environment, are important factors in achieving better outcomes in tissue remodeling. This concept has been termed “dynamic reciprocity” [110]. With a further understanding of the biological complexity and the physicochemical nature of native tissue, the appropriate design of new smart biomaterials will be possible by active interdisciplinary collaboration, bringing together polymer chemists, biomaterials scientists, tissue engineers, and clinicians (Figure 6).

## 6. Conclusions

The performance of the current treatments for long urethral strictures remains suboptimal due to the shortage of tissue sources, the significant donor site morbidity, or the inability to completely restore the structure and function of the host. While non-degradable polymers have not met the clinical performance requirements because of the potential risk of migration, encrustation, and ultimate narrowing, tissue-engineered solutions based on biodegradable polymers have emerged as potential alternatives for successful recovery, and therefore function, of the urethral structure. Biological scaffolds have shown utility to repair urethral defects, but they display relative mechanical inferiority and their degradability cannot be easily adjusted. On the other hand, synthetic scaffolds have highly controllable chemical, mechanical, and structural characteristics, but proper reproduction of the native extracellular environment remains a challenge. 

This suggests that combinations of natural and synthetic polymers could provide synergy with a controllable degradation rate, mechanical compliance, and supportive of formation of different tissue layers and vascularization. It is important to stress that a successful urethral scaffold should maintain clinical performance (e.g. mechanical function) following implantation, and synchronize with the preclinical performance requirements (e.g. degradation rate, ability to duplicate structure, and integrate into the host). This can potentially be achieved by controlled degradation matching regrowth of healthy tissues. Functional and smart polymeric scaffolds have the potential to better mimic the native tissue and may help tissue engineered solutions to meet the pre-clinical and clinical performance requirements in the future.
